# Supplementary material for: Multiclass arrhythmia classification using multimodal smartwatch photoplethysmography signals collected in real-life settings
Source: Res Sq. 2024 Dec 13:rs.3.rs-5463126. Preprint. [Version 1] doi: 10.21203/rs.3.rs-5463126/v1 (PMC11661413; doi:10.21203/rs.3.rs-5463126/v1)
Supplement: Supplement 1 [file NIHPPRS5463126V1-supplement-1.pdf]

Supplementary Files

This is a list of supplementary files associated with this preprint. Click to download.

- supplementaryver0520241104.docx
